# Supplementary material for: Somatic NGS Analysis of DNA Damage Response (DDR) Genes ATM, MRE11A, RAD50, NBN, and ATR in Locally Advanced Rectal Cancer Treated with Neoadjuvant Chemo-Radiotherapy
Source: Biomedicines. 2022 Dec 13;10(12):3247. doi: 10.3390/biomedicines10123247 (PMC9775018; doi:10.3390/biomedicines10123247)
Supplement: Supplementary file 1 [file biomedicines-10-03247-s001.zip › biomedicines-2022601-supplementary.pdf]

Supplementary Materials

Panel design: Custom Panel were designed with Ampliseq Designer V.7.21 (https://ampliseq.com/protected/startPage.action). Specific panel features are listened in Table S1: 5 genes, 492 amplicons (Pool1: 246 amplicons, Pool2: 246 amplicons) with size range 125-175 bp and size: 38.73 Kb;

Table S1. Ion Ampliseq™ Panel Specifications.

Gene_Symbol	RefSeq	Chr	Chr_Start	Chr_End	Target (bp)	Missed (bp)	covered (%)
MRE11	*NM_005591.3*	chr11	94153277	94226023	4082	0	100
ATM	*NM_000051.3*	chr11	108098341	108236298	15791	0	100
ATR	*NM_001184.3*	chr3	142168222	142297568	9234	0	100
RAD50	*NM_005732.3*	chr5	131893007	131978077	6628	0	100
NBN	*NM_002485.4*	chr8	90947762	90996829	4118	0	100

Abbreviations: RefSeq, Reference Sequence; (https://www.ncbi.nlm.nih.gov/refseq/); chr, chromosome. Genome: hg19.

NGS Amplification and Sequencing Conditions: Multiplex amplification was performed with 10 ng of DNA using Ion AmpliSeq Library Kit Plus according to the manufacturer's protocol, libraries were carried out by emulsion PCR using Ion PGM™ (Personal Genome Machine) Hi-Q™ (Thermo Fisher Scientific, Carlsbad, CA, USA) View OT2 Kit (Thermo Fisher Scientific, Carlsbad, CA, USA) on Ion OneTouch 2 Instrument (Thermo Fisher Scientific, Carlsbad, CA, USA) and the Ion OneTouch ES (Enrichment System) (Thermo Fisher Scientific, Carlsbad, CA, USA) to produce high-quality Ion Sphere™ particles for use in combination with the Ion PGM™ Hi-Q™ View Sequencing Kit (Thermo Fisher Scientific, Carlsbad, CA, USA).

Analysis Filters Applied: Exclusion of intronic regions, except for the AG/GT splice sites, recurrent variants that likely are sequencing or homopolymer errors that often occur at VAF <5%, and single nucleotide polymorphisms (SNPs) present in the healthy population. Therefore, somatic mutations were considered if the variant allele was present in more than 2% of the reads, considering a minimum coverage depth of 1000×. Called variants were imported, annotated, and filtered in Ion Reporter Server (IRS). To reduce the affect of deamination in low-quality FFPEs transition/ transversion ratio (Ts/Tv) was calculated by bioinformatics tools in IRS and all identified variants was filtered. The identified variants were filtered for allele fraction (VAF>5%), mapping quality, strand ratio

ddPCR Primer Design and Amplification Conditions: PCR primers were designed to detect somatic single-nucleotide variants in cancer mutations. A mutation- or wild-type-specific reverse primer is paired with a common target forward primer. We designed the region of primers to be around 20 bps in length with the SNP as the last base pair on the 3'. The primers were designed to produce small amplicons length about 90 base pair.

The thermal conditions were as follows: one cycle of enzyme activation at 95°C for 5 minutes; denaturation at 95 °C for 30 seconds, immediately followed by annealing/extension 60° for 1 minute for a total of 40 cycles; a final droplet stabilization step at 4 °C for 5 minutes; 90°C for 5 minutes; and an extended 4°C hold. After thermal cycling the plate was transferred and read by Bio-rad QX200 Droplet reader.

Amplitude files were generate using the Bio-Rad droplet reader software QuantaSoft version 1.7.4. The resulting single-color dye fluorescent signal has a direct relationship to size of the amplicon product.
